# Partners in Mischief: Functional Networks of Heat Shock Proteins of *Plasmodium falciparum* and Their Influence on Parasite Virulence

**DOI:** 10.3390/biom9070295

**Published:** 2019-07-23

**Authors:** Michael O. Daniyan, Jude M. Przyborski, Addmore Shonhai

**Affiliations:** 1 Department of Pharmacology, Faculty of Pharmacy, Obafemi Awolowo University, Ile-Ife, Osun State 220005, Nigeria; 2 Center of Infectious Diseases, Parasitology, University of Heidelberg Medical School, INF324, 69120 Heidelberg, Germany; 3 Department of Biochemistry, School of Mathematical & Natural Sciences, University of Venda, P. Bag X5050, Thohoyandou 0950, South Africa

**Keywords:** *Plasmodium falciparum*, heat shock proteins, exportome, functional interplay, chaperone, co-chaperone

## Abstract

The survival of the human malaria parasite *Plasmodium falciparum* under the physiologically distinct environments associated with their development in the cold-blooded invertebrate mosquito vectors and the warm-blooded vertebrate human host requires a genome that caters to adaptability. To this end, a robust stress response system coupled to an efficient protein quality control system are essential features of the parasite. Heat shock proteins constitute the main molecular chaperone system of the cell, accounting for approximately two percent of the malaria genome. Some heat shock proteins of parasites constitute a large part (5%) of the ‘exportome’ (parasite proteins that are exported to the infected host erythrocyte) that modify the host cell, promoting its cyto-adherence. In light of their importance in protein folding and refolding, and thus the survival of the parasite, heat shock proteins of *P. falciparum* have been a major subject of study. Emerging evidence points to their role not only being cyto-protection of the parasite, as they are also implicated in regulating parasite virulence. In undertaking their roles, heat shock proteins operate in networks that involve not only partners of parasite origin, but also potentially functionally associate with human proteins to facilitate parasite survival and pathogenicity. This review seeks to highlight these interplays and their roles in parasite pathogenicity. We further discuss the prospects of targeting the parasite heat shock protein network towards the developments of alternative antimalarial chemotherapies.

## 1. Introduction

Malaria accounts for an estimated 435,000 deaths annually [1]. The victims of the disease are mainly children under the age of five and pregnant women [2]. Malaria is caused by protozoan members of the genus *Plasmodium* of which *P. falciparum* causes the most lethal form of the infection [3]. Malaria is spread by infected female *Anopheles* mosquitoes upon biting humans during a blood meal. The mosquito inoculates the human host with sporozoites. Upon their entry into the human body, malaria parasites initially multiply in the liver cells, which thereafter rupture to release merozoites that then invade red blood cells [3]. The invasion of red blood cells by merozoites is rapid (accomplished in approximately less than two minutes following the release of merozoites) [4]. This rapid invasion of red blood cells by the merozoites reduces the time they spend within the extracellular space, thus reducing their exposure to host immunity [4]. The invasion of host cells by *P. falciparum* relies on the parasite’s acto-myosin motor system which enables traction of the parasite into the host cells [5]. It is at the blood stage that clinical malaria symptoms develop. 

Obligate intracellular parasites are thought to possess substantially “eroded” genomes because of the convenience associated with living in the host environment [6]. Some of these genetic erosions lead to inaccurate translation of the genetic code, leading to the production of mutated proteins [6]. For this reason, compared to their free-living counterparts, obligate parasites produce higher proportions of aggregation prone proteins [7]. A recent study reported that the proteome of *P. falciparum* is characterized by an enriched content of glutamine (Q) and asparagine (N) motifs [8]. For this reason, the same study observed that nearly 10% of the parasite proteome is involved in amyloid-like associations [8]. Altogether, these observations suggest that *P. falciparum,* along with other obligate parasites, needs to employ robust protein quality control machinery in order to deal with living in the host environment.

**Figure 1:**
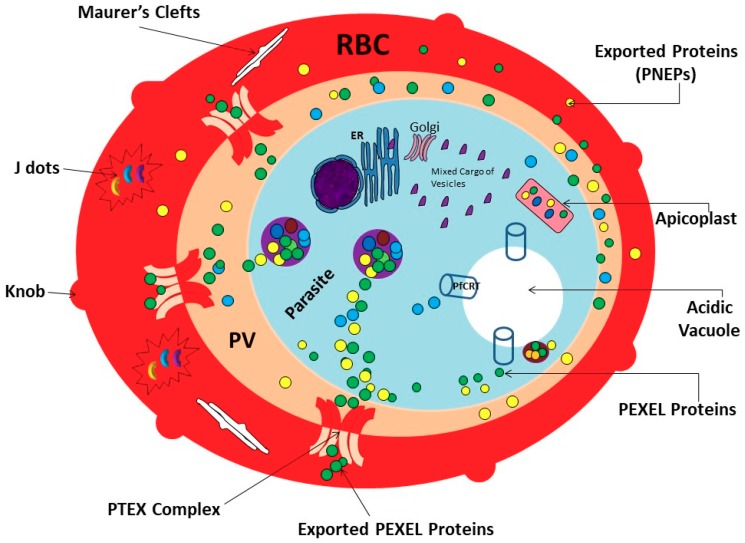
***P. falciparum* protein export pathways**. ER—endoplasmic reticulum; PV—parasitophorous vacuole; PTEX—*Plasmodium* translocon of exported proteins; RBC—red blood cell; PEXEL—proteins export element; PNEPs—PEXEL-negative export proteins; PfCRT—*Plasmodium falciparum* chloroquine transporter. The figure was adapted from [11,14,15].

Remarkably, the parasite is endowed with a genome that allows it to adapt to physiological changes that are associated with a stint in the mosquito vector followed by its development in the host under various physiologically distinct growth stages [9]. Heat shock proteins constitute the cell’s protein folding machinery and are generally upregulated during cellular stress [10]. Perhaps it is not surprising that nearly 2% of the parasite genome encodes for various members of the heat shock protein family. In addition, heat shock proteins of the parasite constitute a large fraction (about 5%) of the nearly 450 proteins of parasite origin predicted to be exported to the infected red blood cells [10,11]. The export of some of these proteins facilitates the modification of the infected host red blood cell, making it cyto-adherent, thus leading to complications linked to malaria (Figure 1) [12,13]. 

Functional versatility is one of the hallmarks of heat shock proteins as they are capable of facilitating protein synthesis and folding while simultaneously titrating misfolded proteins to refold them or to degrade them should they be damaged beyond repair [16]. It has been suggested that expression of parasite heat shock proteins is linked to progression of clinical malaria [17]. What is becoming evident is that these proteins are not only important in providing cyto-protection to the parasite during its development in the host, but that heat shock proteins are particularly important in host invasion and further promote pathogenicity. For example, ablation of the gene encoding for a small heat shock protein, Hsp20 of *Plasmodium berghei*, reduced motility of the parasite sporozoites, thus compromising their ability to infect host liver cells [16]. *P. berghei Hsp20*-deficient sporozoites were reported to exhibit compromised motility and aberrant trajectories when subjected to gliding assays on glass slides [18]. This suggests that apart from their role in proteostasis, small heat shock proteins may regulate parasite motility by modulating its acto-myosin motor system. 

Several efforts to eliminate the disease have been implemented, including control of mosquito vectors, treatment, and efforts towards finding an effective vaccine [19,20]. One of the challenges that are setting back malaria control and elimination efforts is drug resistance by the parasite [21]. For the treatment of uncomplicated malaria, the World Health Organization (WHO) recommends the use of the fast-acting drug artemisinin in combination with a partner drug. The rationale is that the fast-acting artemisinin protects the partner drug against parasite resistance. However, the emergence of resistance against artemisinin continues to be reported especially in parts of Southeast Asia [22]. Thus, the current first line antimalarial drug, artemisinin, now faces reduced efficacy, hence the need for identification of novel antimalarial drugs and their respective targets. It should be noted that generally antimalarial drugs cause oxidative stress, which in turn induces parasite heat shock protein expression, and the cyto-protective functions of the upregulated parasite heat shock proteins augment antimalarial drug resistance [23]. In addition, it is important to understand the parasite biology and to decipher how it develops resistance against antimalarial chemotherapeutic agents. In light of their implication in parasite drug resistance and their ubiquity in the cell, heat shock proteins of *P. falciparum* remain a focus of research interest.

The current review focuses on highlighting recent advances in the functional features of *P. falciparum* heat shock proteins with respect to their roles (including as folding and refolding agents) in the development and pathogenicity of malaria. In addition, we discuss not only their individual roles in the development of the parasite, but also highlight their functional interplay. The prospects of targeting parasite heat shock proteins in the fight against malaria has been previously proposed [14,24,25]. Recent studies highlighting the role of heat shock proteins in the development of drug resistance by the parasite has justified this approach [21,26,27,28]. However, their role in parasite drug resistance make them amenable to antimalarial targeting, especially in combination therapies.

## 2. Functional Interplay of Plasmodial Heat Shock Proteins

The continuous survival and development of the malaria parasite is intrinsically dependent on its ubiquitously expressed molecular chaperones [29,30]. The Hsp90, Hsp70, and Hsp40 members of the heat shock protein family are important for parasite survival and several of them are essential. Molecular chaperones play an important role during de novo protein synthesis and facilitate the subsequent trafficking of proteins to desired destinations, and they further oversee the folding of proteins to their native three-dimensional conformations, as well as facilitate assembly of multi-protein complexes [31,32,33]. Furthermore, molecular chaperones maintain surveillance on cellular protein quality, channeling irreparably damaged proteins for timely degradation [31,33]. While some molecular chaperones are constitutively expressed [34,35,36], others are stress-induced [37]. 

Molecular chaperones are known to occur in functional networks in which their regulatory elements (co-chaperones) play an important role [38,39,40]. One of the most distinct molecular chaperone systems of the parasite involves the Hsp90 and Hsp70 pathway [41,42]. *P. falciparum* cytosol/nuclear localized Hsp90 (PfHsp90; PF3D7_0708400) and Hsp70-1 (PfHsp70-1; PF3D7_0818900) function independently as well as in partnership with one another [43]. On the other hand, while some members of Hsp40 family are known to also function as independent chaperones whose role in this respect is limited to suppression of protein misfolding [44], they also largely serve as co-chaperones of Hsp70 partners [45,46]. Hsp40s serve as substrate scanners, whose main task is to detect partially misfolded peptides, binding onto them first before channelling them to Hsp70 for refolding [45,46]. Upon handing over the peptide substrates to Hsp70, Hsp40 simultaneously stimulates the otherwise rate-limiting ATP hydrolysis of Hsp70 [45,46]. A bioinformatics-based prediction approach was previously used to map out functional partners of the Hsp90–Hsp70 pathway of the parasite [47]. This was followed up by biochemical assays validating the functional players involved in this pathway, including the associated co-chaperones [43,44,48,49]. The functional interplay between *P. falciparum* Hsp70 (PfHsp70; PF3D7_0818900) and PfHsp90 (PF3D7_0708400) both of which are cytosol and nuclear localized is made possible through an adaptor molecule, Hsp70–Hsp90 organizing protein (PfHop; PF3D7_1434300) [43,48]. In addition, *P. falciparum* Hsp70-x (PfHsp70-x; PF3D7_0831700) was found in complex with two of the parasite exported type II Hsp40 co-chaperones, PFA660 (PF3D7_0113700) and PFE55c (PF3D7_0501100), in the infected host red blood cell [50]. Notably, both PFA660 (PF3D7_0113700) and PFE55c (PF3D7_0501100) are trafficked from the parasite cell to the host red blood cell. This suggests that malarial proteins that are destined to the host red blood cell probably require refolding in this foreign environment by an ‘’emissary’’ set of molecular chaperones that originate from the parasite cell. Indeed, some recombinant parasite proteins expressed in foreign environments such as *Escherichia coli* cells have benefitted with respect to enhanced expression and folded quality by co-expressing them with Hsp70 chaperones of *P. falciparum* origin [51,52]. Altogether, this suggests that Hsp70 is a functionally specialized protein across species. Reviews on the general functions of plasmodial Hsp90, Hsp70, and Hsp40 have been previously presented [14,39,41,53]. Here we highlight current findings on heat shock proteins of *P. falciparum* and the importance of their partnerships in the survival and pathogenicity of the parasite.

### 2.1. Plasmodium falciparum Hsp90 (PfHsp90)

Malaria parasites encode four Hsp90 homologues, which can be assigned based on bioinformatics analysis, immunolocalization, and expression of reporter tagged transgenes to four sub-cellular locations: cytoplasm, mitochondrion, endoplasmic reticulum (ER), and apicoplast [16]. All four homologues are highly similar amongst themselves, and also share a high degree of similarity to Hsp90s from other systems [30]. Hsp90 is known to be expressed at the ring, trophozoite, and schizont stages of the parasite life cycle [54] and constitutes about 2% of the parasite proteome [30]. In general, Hsp90 is involved in facilitating folding of molecules involved in signal transduction such as kinases and steroid hormone receptors [55]. Of the two homologous isoform Hsp90s localized in the cytosol, it is the inducible isoform, PfHsp90 (PF3D7_0708400), which has been more extensively studied compared to its constitutive cytosolic counterpart. PfHsp90 (PF3D7_0708400) is upregulated by general physiological stress, including antimalarial drug-induced stress [56]. It has further been proposed that inhibitors of Hsp90 reverse drug resistance in fungi [57]. Indeed, inhibition of Hsp90 has been found to enhance the antiplasmodial activity of aminoalcohol-carbazoles [58]. As Hsp90 expression is associated with both the development of clinical malaria and parasite drug resistance to artemisinin [28], targeting Hsp90 in antimalarial combination therapies is a promising approach. However, it is not yet clear if Hsp90 inhibitors of *P. falciparum* target all four Hsp90 homologues of the parasite [58]. In the event the inhibitors target all four proteins this would present a “quadruple whammy” against the parasite. Binding pockets of Hsp90 are highly conserved across *Plasmodium* species, suggesting that their inhibition is stringent and this reduces prospects for drug resistance. Geldanamysin (GA) has been shown to inhibit PfHsp90 (PF3D7_0708400) and reportedly killed parasites at the blood stage with median inhibitory concentration (IC_50_) of 20 nM, compared to the IC_50_ of 15 nM for chloroquine (CQ) [59]. GA was effective against CQ-resistant and CQ-susceptible parasites, and the combination of the two drugs acted synergistically against parasites maintained at the blood stages [59]. Even more encouraging is that a follow-up study established that Hsp90 inhibitors acted against parasites at the early stages of growth [60]. This suggests that inhibitors of Hsp90 may act before parasite drug resistance develops. In other parasites, Hsp90 has been described as a regulator of stage differentiation by acting as a cellular thermosensor [61]. It would be tempting to investigate whether such a function is also conserved in malaria parasites, possibly during periods of changing environmental conditions such as ingestion by the mosquito and subsequent gametocyte activation and/or during sporozoite injection and subsequent invasion of liver cells. 

### 2.2. Plasmodium falciparum Hsp70 (PfHsp70)

All *Plasmodium spp*. encode at least five Hsp70 homologues, referred to as Hsp70-1 (PF3D7_0818900), Hsp70-2 (PF3D7_0917900), Hsp70-3 (PF3D7_113400), Hsp70-y (MAL13P1.540), and Hsp70-z (PF3D7_0708800). However, members of the *Laverania* subgenus encode one additional homologue, Hsp70-x (PF3D7_0831700) [39,62]. Hsp70-1, -2, -3, and Hsp70-x exhibit features of “classical” Hsp70, represented by *E. coli* DnaK, whereas Hsp70-y and Hsp70-z are members of the Hsp110 family [62]. Hsp110 members are structurally larger members of the Hsp70 family and possess acidic extensions in their C-terminal domain [63]. Apart from their role as independent molecular chaperones, they are regarded as nucleotide exchange factors (NEFs) of their canonical Hsp70 counterparts [63].

PfHsp70-1 is the parasite cytosolic Hsp70, characterized by a C-terminal -EEVD motif required for formation of multi-chaperone complexes, likely via interaction with co-chaperones such as PfHop [42,48]. Hsp70-2 is a binding immunoglobin protein/glucose regulated protein 78 (BiP/GRP78) homologue, characterized by an ER signal sequence and C-terminal ER retrieval sequence, -SDEL [64]. Although yet to be experimentally demonstrated, it is thought that Hsp70-3 resides in the mitochondrion by virtue of a predicted N-terminal mitochondrial targeting sequence. Hsp70-y and Hsp70-z are believed to be NEFs for Hsp70-2 and Hsp70-1, respectively [65,66]. Hsp70-y thus contains an N-terminal ER-type signal sequence and an additional C-terminal –KDEL, ER retrieval sequence. Although previous reports noted that Hsp70-y also contained an N-terminal apicoplast transit peptide, a later study demonstrated that this signal was overridden by the -KDEL sequence, and thus it is likely that Hsp70-y is a bona fide protein of the ER lumen [62,67]. The mitochondrial Hsp70-3 is likely to rely on the NEF function of a Mge1p/GrpE homologue (encoded by PF3D7_1124700), which similarly contains an N-terminal mitochondrial targeting sequence. 

Hsp70-x is secreted into the parasitophorous vacuole (PV), and is also partially exported to the host erythrocyte, where it is suggested to play critical roles in host cell modification (see Section 3) [40,68,69]. Hsp70-x possesses EEVN residues at its C-terminus [62]. This has led to speculation that Hsp70-x may associate with co-chaperones resident in the infected erythrocyte such as human Hop. A recent study showed that the EEVN residues were important for the direct interaction between PfHsp70-x and human Hop *in vitro* [70]. However, it remains to be validated if this interaction takes place *in vivo* (for more on Hsp70-x, its interactions and role in virulence, see Section 3 below). 

Malaria parasites, in comparison to humans and even other parasites, encode a low number of Hsp70s, and it has been suggested that the functional valence of each parasite Hsp70 is expanded by their co-chaperones such as the Hsp40 members [29]. Perhaps supporting evidence for this comes from the fact that the Hsp40 complement of malaria parasites is expanded. For example, while the *P. falciparum* genome encodes for only six Hsp70s, it encodes for at least 49 Hsp40-like proteins [46,53]. Alternatively, it is feasible that differential splicing may lead to expression of various Hsp70 isoforms. However, experimental evidence for this is so far lacking. Unusually, the parasite does not encode a constitutive form (heat shock cognate 70; Hsc70). In other systems, this protein is constitutively expressed and involved in normal cellular proteostasis, while Hsp70 is upregulated upon encountering physiological insult. Thus, Hsp70-1 is constitutively expressed by parasites, and is also upregulated upon heat shock, hence it combines both house-keeping and stress response roles [29]. Similarly, Hsp70-2 and Hsp70-z are upregulated under heat shock conditions, but it is unknown how the remaining Hsp70s react under these conditions [64,65]. 

### 2.3. Plasmodium falciparum Hsp40 (PfHsp40)

Although *P. falciparum* encodes only a minimal set of Hsp70 proteins compared to other organisms, the Hsp40/DnaJ family is extremely well represented, with at least 49 members of this family in the 3D7 strain [46,53]. Even more surprising is the fact that of these 49, almost half are predicted to be transported from the parasite to the human host cell [53,71]. Hsp40s can be classified into type I to IV based on the canonical domains (J-domain, [72], GF-domain [73,74], cysteine-rich zinc binding domain [75], and C-terminal domain [76,77]) present in each. Type I has all domains present, type II has all but lacks the zinc binding domain, type III has just the J–domain, while type IV has the J-like domain with a corrupted non-conserved HPD motif (a highly conserved histidine, proline, and aspartic acid tripeptide of residues) [46,72]. Several of the exported DnaJ proteins are found only in *P. falciparum* (and closely related members of the *Laverani*a subgenus), but not in other *Plasmodium* species infecting either humans, or other hosts, suggesting an important role in the intra-erythrocytic life cycle and survival of *P. falciparum* specifically [78]. As one of the characteristics of *P. falciparum* compared to other species is the presence, on the surface of the infected host cell, of parasite encoded proteins involved in evasion of the host immune response (such as *P. falciparum* erythrocyte membrane protein 1 (PfEMP1)), it was suggested that the exported DnaJ proteins may be involved in transport of such proteins through the host cytosol to the erythrocyte plasma membrane [79,80,81]. Knockout studies support this hypothesis, and demonstrated that, while DnaJ proteins restricted to the parasite cytoplasm appear to be largely essential for blood-stage growth, a number of the exported DnaJs (including PF3D7_0201800, and PF3D7_0501100) are not essential for parasite survival (Table 1), but appear important for host cell modification [13,82]. However, there are exported DnaJs on the other hand that do seem to be essential (Table 1), as it was not possible to isolate knockout mutants [13,82]. Interpreting the importance of these results is slightly impaired by the possibility of functional redundancy between closely related exported Hsp40s, and the lack of a genetic system which would rapidly allow knockout of multiple *dnaj* copies in a single parasite. 

In the more genetically tractable *P. berghei* system, inactivation of a number of both exported and parasite internal DnaJs led to a variety of growth phenotypes in a pooled-knockout approach, with some genes being resistant to inactivation, while some generated slow growing mutants, and some appearing to have no effect on parasite growth [83]. It must be noted that this study only used parasite proliferation rate as a screening phenotype, and thus any less dramatic effects on parasite fitness may have been overlooked. Additionally, as the same study was conducted using a murine malaria model, the observed slow parasite growth may have been the result of not only the direct effect on parasite fitness, but also of changes at the host–parasite interface as well as the host immune response. 

PfHsp70-x (PF3D7_0831700) is exported to the host erythrocyte where it appears to act in concert with two co-exported DnaJs (PF3D7_0113700/PFA660 and PF3D7_0501100/PFE55) of parasitic origin [40,44,50]. However, other species of *Plasmodium* do not encode for an exported *Hsp70*, and yet they encode for DnaJ-like proteins that are predicted to be exported to the erythrocyte. As DnaJ proteins serve as functional regulators of Hsp70s, this begs the question: “What could they be doing out there?” Results from the *P. falciparum* system support a physical and functional interaction between exported DnaJ proteins and resident host cell Hsp70 [38,44], and it is not hard to conceive that a similar interaction may take place in other species lacking their “own” exported Hsp70. Unusually, most of the parasite DnaJ-like proteins exported to the host erythrocyte contain a J-domain which is unlikely to functionally bind to Hsp70 as it contains a mutation within the HPD motif thought to be essential for DnaJ/Hsp70 functional interactions [46]. That type IV (those possessing a corrupted HPD motif) exported Hsp40s do play a role in host cell modification is evidenced by the fact that a knockout of the type IV exported DnaJ protein, ring-infected erythrocyte surface antigen (RESA), resulted in reduction of both host red blood cell rigidity and parasite resistance to heat shock [84]. Also, like RESA, the rest of the type IV DnaJ proteins may not act as typical co-chaperones and instead mediate their function by directly binding to the host cytoskeleton. For a more thorough discussion of the role of exported Hsp40s in host cell modification and virulence, see Section 3 below. The structure–function features of parasite DnaJ members of plasmodial Hsp40 proteins are summarized in Table 1.

## 3. Roles of Plasmodial Heat Shock Protein Functional Interplay in Parasite Virulence

Factors that could determine the level of virulence of a parasite in a host, and hence the severity of infection and the rate of host morbidity, include the presence of co-infecting parasites, either related or unrelated, and the competition among them [86,87]. Other factors are heterogeneity in host genetic susceptibility and dose of initial inoculum [88], the host red cell environment [89], the rate of reproduction and transmission to another host [90,91], the ability of the parasite to maintain balance between longevity and fecundity, the rapidity of damage done to the host, and the length of time the host can continue to supply needed nutrients to keep the parasite alive [86].

The critical roles of exported proteins in parasite survival and virulence through their ability to promote cell rigidity and adhesion, and generation of new trafficking pathways within the infected erythrocytes, have been reviewed [13,14,15,92]. These reviews underscored the importance of these proteins in malaria pathology and their potentials as drug targets. A number of heat shock proteins are involved in host cell modification, and thus virulence. To gain access to the host cell, parasite proteins must traffic across the membrane of the parasitophorous vacuole (PVM). A key factor in this process is the so-called *Plasmodium* translocon of exported proteins (PTEX), a multi-protein complex which sits in the PVM and is a conduit for protein export [93,94]. One important component of this complex is Hsp101, a member of the AAA+ ATPase family. Downregulation, or functional interruption, of this protein leads to a block of protein export in the lumen of the parasitophorous vacuole, and subsequent parasite death [95,96]. As unfolding is required for protein transport through the PTEX [95,97,98], it is likely that Hsp101 fulfils this essential function [97]. Several studies show an association of PTEX with Hsp70-x, and this functional interaction may be required for regulation of Hsp101 activity [79,98]. 

The major parasite derived ligand for cyto-adherence, and thus the pathology associated with *P. falciparum* infection, is PfEMP1, which is synthesized in the parasite and trafficked to the surface of the infected host cell. Proper presentation of PfEMP1 is dependent on several further parasite-derived factors such as the knob-associated histidine rich protein (KAHRP), which is involved in the generation of electron dense structures below the surface of the host erythrocyte, referred to as knobs [79,99]. Knobs act as a platform for PfEMP1 presentation, and parasites lacking KAHRP show reduced cyto-adherence under physiological flow conditions [79,99]. A number of parasite heat shock proteins, including PFB0106c, MAL7P1.172, PF13_0076, PF14_0758, MAL7P1.171, PF10_0025, PFD1170c, and PF10_0381, have been shown to have an effect either on presentation of PfEMP1, stiffness of the host cell, or correct assembly of knobs [13]. Parasites with a deletion in PF3D7_1039100 show both reduced numbers of knobs and cyto-adherence [12]. Also, as previously mentioned, a knockout of RESA (PF3D7_1012200) shows reduced rigidity, while a knockout of PF3D7_0220100 shows an increase in host cell rigidity and cyto-adherence [12,14]. Two independent studies suggest that a deletion mutant of *Hsp70-x* led to reduced rigidity of the infected host erythrocyte, and the same cells exhibited a drop in cyto-adherence [68,69]. Apart from Hsp70-x being reportedly exported to the host erythrocyte where it occurs in complex with PFE55 and PFA660, its direct functional association with PFA660 has been demonstrated [40,44,50,85]. Thus, Hsp70-x may act in concert with exported Hsp40s to support parasite virulence. Although it is possible to delete the gene encoding Hsp70-x under cell culture conditions, genomic studies of patient isolates suggest that, although neighboring genes can be lost by chromosomal rearrangement, *Hsp70-x* deletions are rare, supporting selective pressure to maintain this gene in vivo [94,100,101]. However, it is clear from the foregoing that the parasite needs to jealously maintain these webs of functional interplay among these proteins to keep up with its virulence activities. 

Based on results from high-throughput studies in the murine malaria parasite *P. berghei*, deletion of a number of other exported Hsp40s leads to slow parasite growth in vivo, further supporting an important role for exported Hsp40s in parasite survival and virulence (Table 1) [13]. Pathogens have been shown to exploit the physiological activities of Hsp90 to further their virulence actions. These activities include formation of a multi-chaperone complex with Hsp70, Hop, and other co-chaperones, involvement in signal transduction and cell cycle control, and change in gene expression in response to environmental stimuli [102,103,104]. These activities support parasite survival, promote erythrocyte structural modification, and encourage resistance and evasion of host immune responses. Also important to parasite virulence is the compensatory upregulation of Hsp90 and its multi-chaperone partners (Hsp70, Hsp27, gp96, etc.) in response to drug inhibition [42,47,96], providing alternative options for survival to parasites under unfavorable conditions. This further demonstrates that functional interplay of Hsp90 with other chaperones and co-chaperones is a critical factor in parasite virulence activity. This compensatory action has enhanced the need for an inhibitor or combination of inhibitors to address the persistent dilemma presented by antimalarial drug resistance. 

## 4. Implications for Therapeutic Intervention

In light of the central role of heat shock proteins in cyto-protection, they have been presented as promising drug targets in non-communicable diseases such as cancer [105] and infectious diseases such as malaria [14,106,107]. Several attributes that heat shock proteins possess make them potential drug targets. Firstly, some of the members of this family are essential, making their inhibition lethal [12,13]. In addition, their occurrence in functional networks means that inhibition of one could lead to deleterious consequences down-stream of their metabolic pathways [47]. However, this is not always the case as some heat shock proteins are also known to take over the function of their inhibited counterparts. For example, inhibition of Hsp90 is known to upregulate Hsp70 expression and this has led to the suggestion that co-targeting both proteins has potential in cancer therapy [108]. While it is not immediately clear that transcriptional regulation of Hsp-encoding genes in humans can be translated to *Plasmodium* parasites, targeting of these proteins is a prospective option in antimalarial drug discovery. However, the ‘Achilles heel’ of targeting heat shock proteins is that their inhibition is linked to reversal of drug resistance to traditional antimalarial drugs (reviewed in [27,106]). Perhaps this is not surprising as some heat shock proteins and enzymes implicated in protein quality control appear to play a role in parasite resistance against antimalarial treatment including artemisinin combination therapy (ACTs) [109]. 

Despite the high sequence conservation amongst Hsp90 homologues of *P. falciparum* relative to their counterparts from humans and other species [17], their small differences may suffice to allow design of inhibitors which specifically target only the parasite’s Hsp90s [110]. It has been demonstrated that PfHsp90 (PF3D7_0708400) can be inhibited by a number of known Hsp90 inhibitors, and that growth of both the blood stages and liver stages is reduced by this treatment [56,59,110]. Among the tested inhibitors are GA, 17-DMAG, ganetespib, harmine, and PU-H71 [59,110]. It has been proposed that the synergistic ability of PU-H71 with CQ may be connected with direct or indirect interaction of PfHsp90 (PF3D7_0708400) with *P. falciparum* CQ resistance transporter (*PfCRT*), suggesting that this inhibitor may abrogate their functional interplay [27,111]. Also, in blood stages, inhibition of Hsp90 function blocks ring to trophozoite transition at a point where the parasite is ramping up its protein synthesis [56]. Furthermore, recent studies have revealed that parasite Hsp70s are upregulated in artemisinin-resistant parasites, and that the Hsp70 locus is found in an area of positive selection pressure related to this resistance phenotype [112,113]. It remains however unclear if Hsp70 has a direct influence on arteminisin tolerance, or whether upregulation is part of a general stress response, possibly initiated by the supposed oxidative stress associated with artemisinin exposure [109]. One study using an activity probe-based approach demonstrated direct binding of artemisinin to both Hsp70-1 and Hsp70-2, but the biological significance of this result is still not clear [114]. A larger expression-profiling study showed upregulation of genes involved in the unfolded protein response upon artemisinin exposure, once again suggesting a link between this phenotype and members of the Hsp70 chaperone family [26]. However, this is yet to be experimentally validated. Nevertheless, as upregulation of Hsp70 appears to at least partly protect the parasite from drug treatment, the possibility exists that a combination therapy composed of both artemisinin and Hsp70 inhibitors may lead to more effective parasite killing. Inhibition of parasite encoded Hsp70 leads to parasite death, and several studies have identified inhibitors (including pyrimidinones) which are at least largely specific for parasite versus host Hsp70s [107,115,116,117]. The peptide antibiotic, polymyxin B, and the green tea constituent, (−)-epigallocatechin-3-gallate (EGCG), have recently both been reported to inhibit PfHsp70-1 (PF3D7_0818900) and PfHsp70-z (PF3D7_0708800) function [116,117]. EGCG exhibited antiplasmodial activity with IC_50_ of 2.9 µM, compared to CQ which registered IC_50_ of 8.5 nM under similar growth conditions [118]. Interestingly, a plant extract enriched in epicatechin, which is structurally closely related to EGCG, also inhibited *P. falciparum* Hsp70 and exhibited antiplasmodial action [119]. This suggests that at least some parasite Hsp70s are essential for normal parasite growth, but these studies have not yet found support from reverse genetics, probably due to the hesitation by researchers to attempt to knock out a gene which is highly likely to be essential. In addition, the functional interplay between Hsp40 and Hsp70 constitute another novel avenue for possible chemotherapeutic intervention awaiting to be explored [14]. 

Studies on *P. falciparum* Hop (PfHop; PF3D7_1434300) observed that although conserved it exhibits a fair degree of sequence variation compared to human Hop (PfHop only shares 38% protein sequence identity with human Hop) [43]. For this reason, efforts to identify inhibitors of the Hsp70–Hop–Hsp90 pathway of the parasite constitute a promising approach towards antimalarial drug design [48]. Interestingly, both polymyxin B and EGCG not only inhibited the ATPase and chaperone functions of the two parasite cytosol–nuclear localized Hsp70 isoforms (PfHsp70-1 and PfHsp70-z), but they also abrogated the functional interaction of the two proteins as well as suppressed interaction of PfHsp70-1 with the co-chaperone, PfHop (PF3D7_1434300) [116,117]. Despite the fact that the binding sites of EGCG and polymyxin are reportedly located in the N-terminal ATPase domain, the two compounds were capable of abrogating interaction of PfHop with PfHsp70-1 which occurs via the C-terminal EEVD motif of the latter [48,116,117]. Similarly, phenylethynesulfonamide (PES), an inhibitor targeting the C-terminal substrate binding domain of Hsp70, has also been shown to abrogate its association with several of its co-chaperones and substrate proteins in cancer cells [118]. This suggests that Hsp70 inhibitors targeting the N-terminal ATPase and C-terminal substrate binding domains, respectively, not only interfere with the primary domain to which they are directly bound but also abrogate the function of remotely positioned motifs of the protein. The consequence of this is that Hsp70 inhibitors interfere with its functional commitments in toto. Interestingly, the same phenomenon was reported for Hsp90 which was inhibited by GA based on a yeast cell free model resulting in the chaperone’s substrate, apoprotein B, being channeled for degradation [120]. This further reiterates that molecular chaperones are attractive drug targets as their inhibition by one compound has varied adverse consequences on their functional network partners and substrate clients. 

As molecular chaperones, heat shock proteins ensure that other parasite proteins, including antimalarial drug targets, maintain their integrity, hence their inhibition would present amplified physiological cost to the parasite [25]. Since several compounds that exhibit antiplasmodial function via targeting heat shock proteins have been identified [107,115,116,117,121], it remains important to assess their bioavailability and safety towards their possible application in antimalarial therapy. It is important that efforts be devoted towards chemically modifying the most promising antiplasmodial compounds targeting heat shock proteins towards enhancing their efficacy and selectivity. Furthermore, of importance to addressing the problem of resistance is the identification of promising inhibitors with multi-target activities, such as those capable of dual inhibition of Hsp90 and Hsp70 in *P. falciparum.*

## 5. Conclusions

The functional interplay of plasmodial proteins is a critical phenomenon that is important to our understanding of the pathogenesis of malaria infection. Critical functions of heat shock proteins, including protein folding, refolding, trafficking, and degradation, as well as formation of knobs on the infected human red blood cell, are not undertaken in isolation, but as cooperate activities, resulting from interplay of functional activities. Also, functional interplay of plasmodial proteins is an important factor in drug resistance, such as through compensatory up-regulation or recruitment of partner proteins following inhibition of their network partner. Therefore, while supporting parasite virulence, functional interplay of plasmodial proteins is a promising avenue for drug intervention.

## Figures and Tables

**Table 1:** Characteristics of Hsp40 homologues.

PlasmoDB ID	Type	Exported?	Knockout			Notes
			[85]	[83], Orthologue	[13]	
PF3D7_0102200 (RESA)	4	N			+	Lower rigidity
PF3D7_0113700 (PFA660)	2	Y	+		-	J-dot [50]
PF3D7_0114000	4	Y	+			
PF3D7_0201700	4	Y	-		+	
PF3D7_0201800 (KAHsp40)	2	Y	+	Non-essential	+	Knob associated [79]
PF3D7_0213100	2	N	-	Non-essential		
PF3D7_0220100	3	Y	-		+	Increased rigidity, binding
PF3D7_0220400	4	Y	+		+	
PF3D7_0409400	1	N	+	Essential		
PF3D7_0500800	4	Y	+	Non-essential/slow		
PF3D7_0501100 (PFE55)	2	Y	-	Non-essential	+	J-dot [50]
PF3D7_0502800	3	N	+			
PF3D7_0523400	3	N	-	Slow		
PF3D7_0626600	3	N	-			
PF3D7_0629200	2	N	+			
PF3D7_0724400	3	N	-			
PF3D7_0806500	3	N	-			
PF3D7_0823800	3	N	-	Slow		
PF3D7_0919100	3	N	-	Essential		
PF3D7_0920100	3	N	-	Slow		
PF3D7_1002800	3	N	+			
PF3D7_1005600	3	N	+	Non-essential		
PF3D7_1038800	3	Y	+		+	
PF3D7_1039100	4	Y	+		+	Reduced knobs and cyto-adherance
PF3D7_1102200	4	Y	+		-	
PF3D7_1108700	2	Y	-	Essential		
PF3D7_1126300	3	N	-			
PF3D7_1136800	3	N	-	Essential		
PF3D7_1143200	4	N	+	Slow		
PF3D7_1149200	4	Y	+		-	
PF3D7_1149500 (RESA2)	4	Y	+		+	
PF3D7_1149600	3	Y	+		+	
PF3D7_1201100	3	Y	+		+	
PF3D7_1211400	2	N	-			
PF3D7_1216900	3	N	-	Essential		
PF3D7_1253000	4	Y	+		+	
PF3D7_1307200	3	N	-	Essential		
PF3D7_1318800	3	N	-	Essential		
PF3D7_1356700	2	N	-			
PF3D7_1401100	4	Y	-			
PF3D7_1413900	2	N	-	Essential		
PF3D7_1437900	1	N	-			
PF3D7_1473200	3	N	-			
PF3D7_0831200	3	Y	-			

Export prediction based on PlasmoDB. For knockout studies, a ‘’+’’ sign shows that a knockout was previously reported, while ‘’-’’ shows that no knockout mutants could be obtained. ‘’Y’’ represents ‘’Yes’’, i.e., acknowledging export of the respective protein, and similarly, ‘’N’’ represents ‘’No’’, for lack of data supporting export. 1, 2, 3, and 4 represent types I, II, III, and IV Hsp40 proteins, respectively. “Slow” means slow growth compared to the wild-type parent. Phenotypes in “notes” column refer to the effect of gene knockout. Figure adapted from [14,46]. References in the table are here given: [13], Maier et al. (2008); [50], Külzer et al. (2010); [79], Acharya et al. (2012); [83], Bushell et al. (2017); [85], Zhang et al. (2018).
